# Exploring Possible Ways to Enhance the Potential and Use of Natural Products through Nanotechnology in the Battle against Biofilms of Foodborne Bacterial Pathogens

**DOI:** 10.3390/pathogens12020270

**Published:** 2023-02-07

**Authors:** Kannappan Arunachalam, Ganesh Prasath Krishnan, Sathya Sethuraman, Sybiya Vasantha Packiavathy Issac Abraham, Swetha Thirukannamangai Krishnan, Aakanksha Venkateswar, Jagadeesan Arunkumar, Chunlei Shi, Davoodbasha MubarakAli

**Affiliations:** 1 MOST-USDA Joint Research Center for Food Safety, Department of Food Science and Technology, School of Agriculture and Biology, and State Key Lab of Microbial Metabolism, Shanghai Jiao Tong University, Shanghai 200240, China; 2 Department of Biotechnology, Sri Venkateswara College of Engineering, Sriperumbudur 602117, Tamil Nadu, India; 3 Department of Biotechnology, Alagappa University, Karaikudi 630002, Tamil Nadu, India; 4 Department of Biotechnology, Karunya Institute of Technology and Sciences, Coimbatore 641114, Tamil Nadu, India; 5 Department of Pediatrics, Ann and Robert H Lurie Children’s Hospital of Chicago, Northwestern University, Chicago, IL 60611, USA; 6 Department Clinical and Translational Sciences, Joan C. Edward School of Medicine, Marshall University, Huntington, WV 25755, USA; 7 School of Life Sciences, B.S. Abdur Rahman Crescent Institute of Science and Technology, Chennai 620048, Tamil Nadu, India

**Keywords:** antibiofilm, biofabrication, foodborne pathogens, nanocarrier, natural preservatives

## Abstract

Biofilms enable pathogenic bacteria to survive in unfavorable environments. As biofilm-forming pathogens can cause rapid food spoilage and recurrent infections in humans, especially their presence in the food industry is problematic. Using chemical disinfectants in the food industry to prevent biofilm formation raises serious health concerns. Further, the ability of biofilm-forming bacterial pathogens to tolerate disinfection procedures questions the traditional treatment methods. Thus, there is a dire need for alternative treatment options targeting bacterial pathogens, especially biofilms. As clean-label products without carcinogenic and hazardous potential, natural compounds with growth and biofilm-inhibiting and biofilm-eradicating potentials have gained popularity as natural preservatives in the food industry. However, the use of these natural preservatives in the food industry is restricted by their poor availability, stability during food processing and storage. Also there is a lack of standardization, and unattractive organoleptic qualities. Nanotechnology is one way to get around these limitations and as well as the use of underutilized bioactives. The use of nanotechnology has several advantages including traversing the biofilm matrix, targeted drug delivery, controlled release, and enhanced bioavailability, bioactivity, and stability. The nanoparticles used in fabricating or encapsulating natural products are considered as an appealing antibiofilm strategy since the nanoparticles enhance the activity of the natural products against biofilms of foodborne bacterial pathogens. Hence, this literature review is intended to provide a comprehensive analysis of the current methods in nanotechnology used for natural products delivery (biofabrication, encapsulation, and nanoemulsion) and also discuss the different promising strategies employed in the recent and past to enhance the inhibition and eradication of foodborne bacterial biofilms.

## 1. Introduction

**Figure 1:**
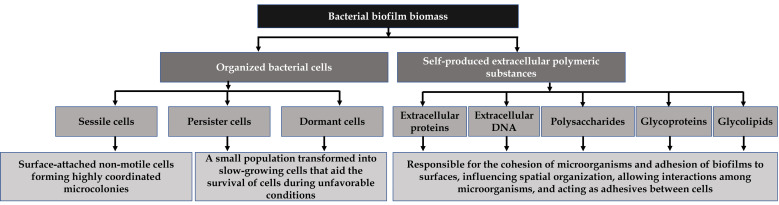
General summary of the composition of bacterial biofilms.

Biofilms are a community of mono or mixed species of sessile bacteria encased in self-produced extracellular polymeric substances, which have physiological characteristics that distinguish them from their planktonic counterparts [1]. Biofilm formation is a complex adaptive mechanism that shields biofilm residents from unfavorable conditions, such as human defenses, disinfectants, and antibiotics [2]. The extracellular polymeric substances produced by the bacteria include proteins, polysaccharides, and extracellular eDNA (Figure 1), which allow the bacteria to communicate and maintain three-dimensional structures [3].

Pathogenic microbial communities in the form of biofilms present challenges for the food industry [4,5]. The almost unavoidable biofilm development on food processing equipment threatens the hygienic quality of the final food products, raising food safety concerns and potentially endangering consumer health [6]. Many approaches have been utilized to overcome the biofilm formation of pathogenic bacteria in food sectors, for instance, with physical, chemical, and mechanical methods [7]. However, the bacteria residing in biofilms are far more resistant to these treatments [8,9].

The potential of natural products and phytocompounds against bacterial pathogens’ biofilm formation and eradicating preformed biofilms has been well studied. Several phytocompounds and other natural products with their exact mechanism of action against bacterial biofilm, especially foodborne pathogens, were recently reviewed in detail by others [10,11,12]. However, using natural products has limitations, like stability and bioavailability, when it comes to their application [13]. Hence, the present review is intended to cover the use of nanoparticles or nanoformulations containing different natural products with biofilm targeting potential against foodborne pathogens published in recent years. The goal is to assess the recent development of new nanotechnology approaches that increase the activity of underutilized bioactive natural compounds. As a result, these substances can be considered for the rational design of highly effective, sophisticated antibiofilm strategies with the potential for clinical and translational studies.

## 2. Significance of Pathogenic Bacteria and Their Biofilms in the Food Industry

According to earlier reports, materials typically found in the food industry, such as Buna-N, Teflon seals, rubber seals, stainless steel, aluminum, and glasses, as well as food-processing units like heating exchangers, cooling towers, conveyor belts, wastewater pipes, etc., can harbor pathogenic microbes like *Escherichia coli*, *Staphylococcus aureus*, *Listeria monocytogenes*, *Campylobacter jejuni*, *Salmonella* spp., *Bacillus cereus*, and *Pseudomonas* spp. in the form of biofilms (Table 1) [6,14].

In food-processing facilities, biofilms have been a critical cause of food deterioration related to economic losses and food safety problems, leading to several outbreaks [6,26]. Every year, numerous foodborne-related outbreaks are documented globally. The financial loss associated with illnesses caused by foodborne pathogens in the United States is estimated to hit $78 billion annually [5,27]. In 2019, the European Union, consisting of 27 member states, reported 5175 foodborne outbreaks, with 49,463 illnesses, 3859 hospitalizations, and 60 deaths [28]. In this context, outbreaks related to *Listeria* and *Salmonella* infections significantly cause the highest number of diseases. These facts prompted researchers worldwide to dive further into the knowledge of elements contributing to biofilm formation and resiliency to create better strategies to limit possible outbreaks and manage illnesses caused by foodborne pathogens. [14,15].

Typically, microbes related to the food industry such as *B. cereus*, Enterohemorrhagic *E. coli*, *L. monocytogenes*, and *S. enterica*, are predominantly present in the mixed biofilm state [7]. Moreover, *B. cereus* forms endospores that can withstand pasteurization conditions and make robust biofilms [29]. The enterotoxin (hemolysin) of *B. cereus* causes various illnesses, namely diarrhea and abdominal pain [7]. This kind of toxin is stably present in foods with higher butterfat content and in High-Temperature-Short-Time pasteurized food products [30]. In the case of enterohemorrhagic *E. coli*, transmission occurs via ingesting contaminated fruits, vegetables, meat, milk, and distilled water. The *E. coli* strain O157:H7 forms thick biofilms on stainless steel and borosilicate glass but not on polypropylene surfaces [31]. It can potentially secrete Shiga toxins (STEC), spawn enterohaemorrhagic gastroenteritis, and cause watery diarrhea and blood in feces [32]. Compared to other foodborne pathogens, *L. monocytogenes* is a deadly pathogen that accounts for the increased mortality rate [33]. The recent incidence in South Africa was recorded as the largest *Listeria* outbreak and attracted the attention of ordinary people to the importance of food safety [34]. In addition, *S. enterica* and *S. aureus* are potential pathogens in biofilm formation and causing infection-mediated diseases. Methicillin-resistant *S. aureus* (MRSA) is prevalent in farm animals and forms biofilms on many animal surfaces [35].

## 3. Natural Products as a Promising Alternate

The emergence of drug-resistant microbes is mushrooming at an alarming rate. Compared with other approaches, using natural bioactives could be a promising alternative that inhibits pathogenic bacterial growth and biofilm formation. Consequently, introducing synthetic or unknown ingredients in a food product to hamper microbial incidences causes consumers to consider the product harmful to their health or body. Therefore, various food additives, including genetically engineered products, artificial sweeteners, taste enhancers, food colorants, and preservatives, contribute to the negative reputation of the product. As a result of this tendency, the food industry is considering natural alternatives to synthetic ingredients.

### 3.1. Natural Bioactives as Preservatives

Several natural sources like plants [13], animals [36], microorganisms [37], bacteriocins [38], and even bacteriophages [39] are being exploited for the identification of bioactive leads against the growth and biofilm formation of foodborne pathogens, which consequently ended up with an overabundance of compounds (Table 2) [37,40]. Phytocompounds are one of the bioactive secondary metabolites that might be used as natural food preservatives. Herbal and plant medicines continue to garner interest as potential therapeutics. A plethora of reports presented the potential of plant bioactives against foodborne pathogens growth and biofilm formation and was reviewed extensively [13,41]. The compounds identified from herbs and spices with good antibacterial and antibiofilm properties are excellent alternatives to chemical ingredients since they are generally considered safe [42,43,44,45]. Investigating plant extracts’ antibacterial and antibiofilm activities may lead to the discovery of novel drugs effective against multidrug-resistant foodborne bacterial strains [46]. As natural preservatives, the phenolic bioactives from herbs and spices also enhance the antioxidant activities against foodborne pathogens [40].

Essential oils are highly concentrated, volatile, hydrophobic chemicals present in a wide variety of plants. The hydroxyl groups of essential oil components, such as those in thymol, carvacrol, and eugenol, react with the phospholipid bilayer of microorganisms resulting in leakage of ions, nucleic acids, and ATP and water imbalance, leading to cell death [40]. The said compounds at sub-MIC were reported to target the biofilm formation of bacterial pathogens in a concentration-dependent manner. Čabarkapa et al. revealed the potential of essential oils rich in thymol in preventing biofilm formation and eradicating preformed *Salmonella enteritidis* biofilms [47]. Similarly, exposure to carvacrol reduces the biofilm formation in different foodborne pathogens, such as *L. monocytogenes* [48], *S. aureus* [49], and *S.* Typhimurium [50].

Furthermore, the bioactives from thyme and rosemary plants reduce the biofilm growth of *L. monocytogenes* and garlic extract can prevent quorum sensing (QS) signaling in multidrug-resistant bacterial pathogens [40,51]. In addition, flavones under the flavonoid class form a complex with the components of the bacterial cell wall and impede cell adherence and proliferation. In this regard, the genes Staphylococcus accessory regulator (*sarA*) and intercellular adhesins (*ica*) are both downregulated by baicalein to suppress the virulence regulation of *S. aureus* [52]. Similarly, it has been shown that the biofilm formation of *S.* Typhimurium (ATCC 14028) was reduced by increasing the concentration of cinnamaldehyde. Moreover, the metabolic activity of preformed biofilms was also inhibited to 39% and 65% upon treatment with cinnamaldehyde at 312 μg/mL and 624 μg/mL, respectively [53].

Similar to plant compounds, reports present the antibiofilm potential of compounds isolated from different microorganisms (Table 2). Very recently, the inhibitory effects of reuterin derived from *Lactobacillus reuteri* (LR 21) isolated from broiler cecum on biofilm formation, quorum sensing, and virulence genes of *Clostridium perfringens* were demonstrated by Xu et al. [54], wherein the reuterin was shown to significantly repressed the surface motility and related biofilm formation of *C. perfringens*. In addition, this compound significantly down-regulated the genes associated with virulence and quorum-sensing expression. D-amino acids isolated from the cell wall of many bacteria have been reported to modulate biofilm formation in *B. subtilis* [55] and *S. aureus* [56].

Glycolipid is a biosurfactant with potential anticancer and antibacterial effects and currently has a wide variety of therapeutic uses [57] including in the pharmaceutical, food, and petroleum sectors. Sophorolipid is one of the glycolipids produced by the yeast *Starmerella bombicola* that has antibacterial and antibiofilm properties against foodborne pathogens such as *C. jejuni*, *E. coli*, *Listeria* spp., and *Salmonella* spp. [58,59,60]. Further, Silveira et al. recently discovered that combining sophorolipid and lactic acid to treat campylobacter cells resulted in an additive interaction and required half of the concentration to treat campylobacters [58].

Many compounds with therapeutic potentials have been discovered in marine algal species such as *Bacillariophyceae* (diatoms), *Chlorophyceae*, *Chrysophyceae*, *Rhodophyceae*, and *Phaeophyceae* [61]. Castillo et al. [62] also found that a commercially manufactured furanone, identical to the extract from *Delisea pulchra*, was effective against Gram-negative *C. jejuni*. AI-2 activity, bacterial motility, and biofilm formation were significantly reduced when coupled with epigallocatechin gallate from green tea and a citric acid extract. Similarly, α-linolenic acid and 1-palmitoyl-sn-glycero-3-phosphocholine isolated from marine microalgae reduced the biofilm formation of *S. aureus* and *E. coli* [63].

**Table 2:** Antibiofilm activity of selected extracts and compounds from different natural resources.

Natural Bioactive and Source	Target Foodborne Pathogen(s)	Mechanism of Action	Reference(s)
**Plant source**			
Essential oil of *Citrus reticulata*	*E. coli* and *S. aureus*	*C. reticulata* essential oil inhibited biofilm formation and eradicated the mature biofilm	[64]
Essential oils components from *Allium sativum* and *Cuminum cyminum*	*S. Typhimurium*	Essential oils eliminated the planktonic and biofilm forms of *S. Typhimurium* by downregulating QS (*sdiA* and *luxS*) and cellulose synthesis (*csgD* and *adrA*) genes	[65]
Essential oil of *Thymus daenensis* and *Satureja hortensis*	*E. coli* O157:H7	Essential oils reduced biofilm formation by downregulating the expression of *luxS* and *pfs* genes	[66]
Essential oil of *Artemisia dracunculus*	*S. Typhimurium* and *S. aureus*	Essential oil inhibited biofilm formation as well as disrupted existing biofilms Downregulation of *luxS* and *pfs* in *S.* Typhimurium and *hld* in *S. aureus* are expected to be the mechanism of action	[67]
Lemongrass essential oil	*L. monocytogenes* and *S. aureus*	In *L. monocytogenes*, an active compound penetrated the biofilm and inhibited its formation In *S. aureus*, biofilm disruption was observed	[68]
Menthol and menthone from peppermint essential oil (PEO) of *Mentha × piperita*	*S. aureus*	Active against biofilm formation in *S. aureus* Moreover, the essential oil effectively killed and eradicated the residents in preformed biofilms	[69]
Thymol from the essential oil of *Thymus zygis*	*L. monocytogenes*	Thymol reduced eDNA release and the expression of enzymes related to biofilm formation In addition, thymol downregulated the expression of biofilm and polysaccharide adhesion-related genes	[70]
Limonene, pinene, terpinene, and myrcene from the essential oil of *Citrus medica*	*L. monocytogenes*	Essential oil components targeted biofilm formation and eradicated the preformed biofilms of *L. monocytogenes*	[71]
Tea catechin extract	MRSA	Tea catechin extract inhibited biofilm formation by downregulating biofilm-related genes such as *fnbA* and *icaBC*	[72]
Exocarp extract of Gingko biloba	MRSA and *S. aureus*	The extract inhibited biofilm formation and preformed biofilms of *S. aureus* Gene expression of *icaA* and *sarA* were lowered after 6 h and *sigB* after 12 h, while upregulation of *icaR* was observed after 12 h	[73]
Peel extract of *Citrus sinensis*	*P. aeruginosa* and *S. aureus*	The peel extract of *C. sinensis* prevented MRSA and ESBL development in the biofilm matrix	[74]
Flesh extract of *Moringa oleifera*	*P. aeruginosa* and *S. aureus*	*M. oleifera* prevented the development of MRSA biofilm matrix *M. oleifera* inhibited the development of single as well as mixed biofilm cultures better than *C. sinensis*	[74]
Root extract of *Veteriveria zizanioides*	*S. aureus*	The methanolic extract prevented the biofilm formation of *S. aureus* Targeted the genes involved in the initial attachment like *clfA* and *fnbAB*	[75]
Barb extract of *Butia odorata*	*S. aureus*	The barb extract reduced the number of biofilm cells	[76]
Ohelo berry extract	*L. monocytogenes*	The extract targeted biofilm formation via downregulation of the global regulator gene *sigB*	[77]
Spice extract from *Syzygium aromaticum* and *Cinnamomum verum*	*L. monocytogenes*	The extract targeted biofilm formation by modulating the release of eDNA In addition, it downregulated the genes responsible for biofilm formation such as *lapB*, *actA*, *flaA*, *prfA*, and *inlA*	[78]
Methanol extract of *Lonicera caerulea var. emphyllocalyx*	*Enteropathogenic E. coli*	The methanolic extract targeted biofilm formation and reduced the spread of biofilm Biofilm-related genes, such as *fliC*, *csgA,* and *fimA*, were downregulated upon treatment with the extract	[79]
Emodin, chrysophanol, and physcion from *Rumex japonicus* extract	*S. aureus*	Inhibition of biofilm formation and removal of biofilm	[80]
Baicalein from *Scutellaria baicalensis* extract	*S. aureus*	Inhibition and disruption of biofilm formation Downregulated staphylococcal enterotoxin A (*sea*) and α-hemolysin (*hla*) levels Downregulated QS-related genes *agrA*, *RNAIII*, and *sarA* as well as *icaA*	[52]
2-hydroxy-4-methoxybenzaldehyde from *Hemidesmus indicus*	MRSA	Inhibition and disruption of biofilms Downregulated virulence factors such as hemolysin, nuclease, lipase and staphyloxanthin Downregulated the global regulator, SigB, required for biofilm formation	[43,81]
Kaempferol from *Glycine max*	*S. aureus*	Inhibited biofilm formation by reducing the expression of sortase (*srtA*) and adhesion-related genes	[82]
Quinic acid	*S. aureus*	Reduction in biofilm biomass Downregulation of *sarA* and upregulation of *agrA* Reduction in the number of sedentary cells on stainless steel	[83]
Naringenin	*S. aureus*	Inhibited biofilm formation by downregulating the expression of *sigB*, *icaA*, *agrA*, and *sarA*.	[84]
Papain from *Carica papaya*	*S. aureus* and *C. jejuni*	Inhibited biofilm formation Degraded the mature biofilm formed on stainless steel	[85]
Phloretin	*L. monocytogenes*	Phloretin targeted biofilm formation by repressing quorum-sensing gene expression	[86]
Pristimerin	*S. aureus*	Inhibition, disruption, and dispersal of biofilm	[87]
Quercetin	*S. Typhimurium* and *S. enteritidis*	Inhibited biofilm formation by downregulating the expression of genes encoding virulence factor (*avrA*, and *hilA*), stress response (*rpoS*), and quorum-sensing (*luxS*)	[88]
Rutin	*E. coli* and *S. aureus*	Inhibition and reduction of biofilm formation Reduction of mono- and multi-species biofilms in a concentration-dependent fashion	[89]
(−)-tetrahydroberberrubine∙acetate (THBA) from *Nandina domestica*	*B. cereus*	Inhibition of biofilm formation and disruption of mature biofilms	[90]
**Animal source**			
BCp12 peptide derived from Milk	* S. aureus *	Inhibited biofilm formation Downregulated the expression of genes related to the QS system, including *agrA*, *agrB*, *agrC*, and *psmB*.	[91]
β-GBP peptide sequence from the *Penaeus vannamei*	* B. subtilis *	Reduced preformed biofilms	[92]
Hc-CATH peptide from sea snake *Hydrophis cyanocinctus* and As-CATH4 and As-CATH5 from *Alligator sinensis*	*A. junii* and * P. mirabilis *	Inhibition, as well as the removal of biofilms	[93]
Manuka Honey	* E. coli * O157:H7	Inhibition, as well as the removal of biofilms	[94]
Apitoxin from *Apis mellifera*	* S. enterica *	Reduced biofilm formation Expression of biofilm and virulence-related genes was different in different strains	[36]
Avarol from *Dysidea avara*	* P. aeruginosa * PAO1	Reduced biofilm formation	[95]
**Bacterial source**			
Peptide AL705	* L. monocytogenes * FBUNT	Reduced biofilm formation by disturbing QS signaling	[96]
Sonorensin from *Bacillus sonorensis MT93*	* S. aureus *	Inhibited biofilm formation by increasing membrane permeability	[97]
Cell-free supernatant of *Lactobacillus curvatus ET31*	* L. monocytogenes *	Inhibition of biofilm formation by targeting *luxS* gene expression However, the cell-free supernatant was not able to remove the preformed biofilm	[38]
**Bacteriophage**			
JK004	* Cronobacter sakazakii *	Removed the biofilm	[98]
vB_STM-2	*S. Typhimurium* (ST-4)	Removed the biofilm Penetrated into EPS and depolymerized EPS	[39]

### 3.2. Challenges in the Use of Natural Preservatives for Preservation

Natural bioactives possess tremendous potential against biofilm-forming bacterial pathogens. However, a plethora of challenges are associated with the usage of natural bioactives as preservatives. Gram-negative bacteria are rich in lipopolysaccharide (LPS), making it tricky for phenolic compounds to penetrate the cell wall [99]. Hence, the catechin monomers from grape seed extract showed increased antibacterial activity towards Gram-positive than Gram-negative bacteria [100]. Similarly, bacteriocins cannot penetrate the LPS of Gram-negative bacteria [101].

The first obstacle to overcome is the extraction of bioactive compounds from natural sources. The extraction technique must be chosen carefully to preserve the quantitative and qualitative properties of the bioactive compounds [102]. Since natural food preservatives need extraction and additional refining procedures, they often increase production costs. Thus, natural food preservatives became more costly than their synthetic counterparts [103].

In the food industry, using natural preservatives may reduce the quality of food and make the food organoleptically unacceptable [104]. The concentration of active compounds greater than the threshold level increases the efficacy loss of food components that alter sensory properties such as taste and smell [105]. Furthermore, spices could provoke intense or pungent aromas in the food. In addition, the storage temperature and heating processes (sterilization and dehydration) are essential factors that induce the active compounds to lose their respective inhibitory activities [105,106].

## 4. Nanotechnology Approach: An Emerging Antibiofilm Platform

Nanotechnology is a promising technology that has the ability to convert an individual particle to one billionth of its original size. The converted particles are nano-sized (1–100 nm), have a large surface area and mass ratio, and are highly reactive, making them completely different from the exact composition of the bulk material [107]. The converted nanoparticles have many advantages, including an increased impact against the target pathogenic microorganisms with multiple functional sites. The exact antibacterial mechanism of nanoparticles has not been entirely elucidated. Many studies have suggested possible mechanisms of action. The absorption of nanoparticles into the cell membrane and the subsequent disintegration are the initial steps involved in the antibacterial mechanism of nanoparticles [108]. Following absorption and disintegration, the cell-penetrating nanoparticles target bacterial growth through intracellular content leakage, generation of reactive oxygen species, impairment of the electron transport system, inactivation of efflux pumps, and most importantly, interference with the enzymatic and metabolic activities of the cell [108].

The discovery and advancement of nanotechnology allow researchers to construct, use, explore, and manipulate nanomaterials in many fields. In the food sector, the application of nanotechnology significantly impacts many areas, such as the production of packaging material, food formulation with enhanced bioavailability, enhancing organoleptic properties, ensuring food safety, and others [109]. It has also become a leading technology in food preservation, particularly in combating foodborne bacterial growth and biofilm formation [110].

Biofilm formation is rapid and spontaneous in the food industry environment compared to medical settings as it deals with nutrient-rich products. For instance, dairy industry instruments can quickly become a source for biofilm formation [111]. Although the inherent antibacterial activity of nanoparticles is well-known, the application of nanoparticles as antibiofilm agents is a relatively new area of research. The ongoing investigations exploring nanoparticles’ interaction with EPS and biofilm residents are still being studied [112,113]. However, a proper understanding of the transport of nanoparticles into the biofilm is vital for designing nanoparticles with customized properties. For reasons like traversing the EPS barrier, enhanced and targeted drug delivery, controlled release, and others, nanoparticles are a viable way to overcome biofilm formation by foodborne pathogens [114,115].

### 4.1. Techniques for Targeting Biofilms through Natural Product Delivery

#### 4.1.1. Biofabrication of Nanoparticles with Antibiofilm Activity Using Surface Functionalization

The surface functionalization of the nanoparticles with phytochemicals or other natural products enhances the bioactivity of the functionalized product. The covalent or non-covalent attachment of the natural product on the surface of the nanoparticles can enhance their solubility, and thereby, its antibiofilm potential. Moreover, the nanoparticle’s penetration enhances the local concentration of the compound inside the biofilm [116]. In this regard, metallic nanoparticles have been vastly exploited (Table 3). Metallic nanoparticles such as gold, silver, copper, and others, synthesized via biological methods (i.e., through plant extracts and natural compounds) frequently have bioactives or compounds in the plant extract as a capping agent [117]. Similar to metallic nanoparticles showing intrinsic activity, these nanoparticles act as a carrier for the bioactives that are physically surface-functionalized. The syntheses of nanoparticles often utilize a salt that is exposed to a redox reaction caused by natural extracts or bioactive compounds under specified pH conditions. The same natural substance is responsible for maintaining the freshly prepared nanoparticle’s surface potential and rendering them with antibacterial/antibiofilm activity after the reduction procedure. As a consequence, the oxidized derivative of the active component caps the nanoparticles [118].

Biofabrication of metallic nanoparticles has gained much interest because of their simple one-step process without producing toxic chemicals. Recently, this technique has been used to assess the biofilm-inhibiting and preformed biofilm-eradicating activity of silver nanoparticles capped with the aqueous extract of *Terminalia catappa* leaf against *L. monocytogenes* [119]. Similarly, silver nanoparticles capped with quercetrin and afzelin curtailed the biofilm formation of *S. enterica* serovar Typhi and *E. coli* [120]. In addition, these biofabricated nanoparticles were reported to rescue animal models like *Caenorhabditis elegans* and zebrafish from bacterial infections, respectively [119,120].

Nanoparticles generally have desirable qualities, such as the ability to change surface characteristics and stability. Meanwhile, selecting phytocompounds and natural products is crucial in determining the size and shape of the nanoparticles. A recent report by Zahoor et al. also demonstrated that amino acids like glutamine, aspartic, and tyrosine are size and shape-controlling agents during silver nanoparticle synthesis [121]. Like silver nanoparticles, the copper nanoparticles synthesized using the glucosides isoquercetin and cassinopin reduced the biofilm formation of MRSA with an effective biofilm inhibitory concentration as low as 1 µg/mL. The prepared nanoparticles exhibited antibiofilm activity against MRSA by altering its cell membrane permeability and surface hydrophobicity [122]. Further, the study’s result revealed that the antibiofilm property was shown by the copper nanoparticle itself and not through its release of copper (Cu(II)) ions. A final method for surface functionalization entails the covalent conjugation of the natural bioactives to the surface of a reactive nanoparticle. This fact was substantiated by the study of Barros et al. [116], wherein the phenol group of curcumin, an active ingredient of the medicinal plant *Curcuma longa*, was linked with the carbohydrate group from the silica nanoparticle through an ester linkage to enhance the antibiofilm property of the silica nanoparticles. Likewise, several studies demonstrated the antibiofilm potential of different metallic nanoparticles such as gold, titanium, zinc, and others, that were surface functionalized with natural extracts or compounds against foodborne bacterial pathogens (Table 3).

**Table 3:** Recent studies on the bioactivity of nanoparticles that were surface functionalized with natural products against the biofilms of foodborne pathogens.

Type of Nanoformulation	Active Agents	Properties of Nanoparticles	Biological Activity against Foodborne Pathogens	Reference
Silver nanoparticles	*Terminalia catappa*	~26–100 nm in size	At sub-MIC concentrations, silver nanoparticles dislodged the preformed biofilms of *L. monocytogenes* and entrenched the biofilm cells. The prepared silver nanoparticles enhanced the life span of infected *C. elegans* by 90%.	[119]
Copper oxide nanoparticles	Leaf extract of *Eucalyptus globulus*	~16.78 nm in size	The tested concentrations inhibited biofilm formation in the range of 44.41 ± 7 to 70.75 ± 8% and 34.41 ± 7 to 62.29 ± 8% in *E. coli* and MRSA, respectively.	[123]
Gold nanoparticles	Caffeine	77.0 ± 5.0 nm in size	At 256 µg/mL, caffeine-loaded gold nanoparticles reduced biofilm formation by 69.55, 63.74, and 64.89% in *S. aureus*, *L. monocytogenes*, and *E. coli*, respectively. At MIC (512 µg/mL), the nanoparticles eradicated preformed biofilms by 63.71 and 77.4% in *E. coli* and *S. aureus*, respectively. It was 79.66% effective against *L. monocytogenes* with an MIC value of 1024 µg/mL. Further, the caffeine-loaded nanoparticles were potent enough to eradicate the biofilm-embedded persister cells of the tested pathogens.	[124]
Gold nanoparticles	Seed Extract of *Trachyspermum ammi*	~24.4 nm in size Shapes appeared to be spherical, spheroid, and a few anisotropies	At 0.5 MIC, the gold nanoparticles reduced biofilm formation by 81 and 73% in *Serratia marcescens* and *L. monocytogenes*, respectively. Gold nanoparticles targeted biofilm formation of the test pathogens by reducing the biofilm-related virulence factors such as cell surface hydrophobicity, motility, and exopolysaccharides. Increased ROS production was expected to play a role in eradicating the preformed biofilms of the tested pathogens.	[125]
Copper oxide nanoparticles	Leaf extract of *Mentha spicata*	Spherical nanoparticles with 36 nm in size	The synthesized copper oxide nanoparticles were superior in inhibiting the biofilm formation of *S. aureus* (98%) compared to *E. coli* (86%) at 100 µg/mL.	[126]
Copper nanoparticles	Cassinopin and isoquercetin from the leaves of *Crotalaria candicans*	Spherical-shaped cassinopin- and isoquercetin-capped nanoparticles were 66 and 69 nm in size, respectively	The synthesized nanoparticles were proficient enough to thwart the biofilm formation of MRSA, *S.* Typhi, and *E. coli,* even at a concentration of 2 µg/mL.	[122]
Silver nanoparticles	Kaempferitrin from *Crotalaria juncea*	Spherical-shaped nanoparticles with an average size of 33 nm and zeta potential of −33.8 mV	Kaempferitrin-loaded silver nanoparticles were able to retard biofilm formation in MRSA. Enhancing the cell membrane permeability in the cells treated with bioactive-loaded silver nanoparticles was suggested to be the antibiofilm mechanism. Further, the silver nanoparticles reduced the bacterial burden by ~1.8-fold in the infected zebrafish with no toxicity.	[127]
Copper nanoparticles	Kaempferitrin from *Crotalaria juncea*	Spherical-shaped nanoparticles with an average size of 56 nm and a zeta potential of −38 mV	Kaempferitrin-loaded copper nanoparticles were able to retard the biofilm formation in MRSA. Enhancing the cell membrane permeability in the cells treated with bioactive-loaded copper nanoparticles is expected to be the possible antibiofilm mechanism. Further, the silver nanoparticles reduced the bacterial burden by ~2-folds in the infected Zebrafish, leaving no toxicity	[127]
Gold nanoparticles	Aerial part extract of *Origanum majoranum*	Spherical-shaped nanoparticles with sizes ranging between 5 and 30 nm	The gold nanoparticles were prepared using L-glutathione. The prepared gold nanoparticles were then conjugated with *O. majoranum* extract. The gold nanoparticles showed antibacterial, antibiofilm, and antioxidant activities. Compared to the extract, the extract-conjugated gold nanoparticles showed antibiofilm activities against *S. aureus* and *E. coli* with inhibition rates of 19.2 and 30%, respectively.	[128]
Zinc oxide nanoparticles	Fruit extract of *Aegle marmelos*	Hexagonal wurtzite-shaped nanoparticle with an average size of 22.5 nm	The zinc oxide nanoparticles coated with *A. marmelos* efficiently inhibited the biofilm formation of *S. aureus* and were validated with confocal microscopic analysis. Further, the use of nanoparticles reduced the pathogen’s hydrophobicity index and enhanced the pathogen’s susceptibility to hydrogen peroxide.	[129]
Silver nanoparticles	*Oscillatoria* sp.	Spherical-shaped nanoparticles with an average size of 10 nm and polydispersity index of 0.58	The synthesized nanoparticles showed potent biofilm inhibition in *S. aureus*, *S. Typhi*, and *E. coli*. The synthesized silver nanoparticles showed reduced toxicity towards the shrimp model *Artemia salina* with an LC50 of 2060 µg/mL.	[130]
Zinc oxide nanoparticles	Leaf extract of *Acacia arabica*	Rod-shaped nanoparticles with an average size of 11.3 nm	The zinc oxide nanoparticles at 0.5 MIC reduced the biofilm formation of *E. coli*, *S. aureus*, and *S. enterica*. Further, the EPS estimation assay revealed that these nanoparticles targeted EPS production to hinder biofilm production in the tested pathogens. The nanoparticles showed no toxicity towards HeLa cells at the tested concentrations.	[131]
Silver nanoparticles	The seed extract of *Nigella sativa*	Spherical-shaped nanoparticles with an average size of 50 nm	The extract-conjugated nanoparticles showed antibiofilm activity against *S. aureus* and *E. coli* with inhibitory rates of 84.92 and 82.84%, respectively.	[132]
Iron oxide nanoparticles	*Eucalyptus globulus* essential oil	Quasi-spherical shaped nanoparticle with an average size of 7.5 ± 2.5 nm	Interfered with the development of biofilm formation in *E. coli* and *S. aureus*. The iron oxide nanoparticles were highly biocompatible for the growth and proliferation of amniotic fluid-derived mesenchymal stem cells.	[133]

#### 4.1.2. Nanoencapsulation of Natural Compounds with Antibiofilm Activity

Arming natural compounds through the nanoencapsulation procedure has several advantages over surface functionalization techniques. First, they enable sustained release of the bioactive compounds, where the drug release profile is governed by the properties of the polymer being used for encapsulation, such as drug–matrix interactions, solubility, diffusion, and biodegradation [134]. Through nanoencapsulation, it is possible to regulate the delivery of natural bioactives to the target site using site-specific stimulants, such as ultrasound, enzymes, pH, and magnetic fields [135,136,137,138]. This functional property aids researchers in exploiting nanocarriers as a portable detection system for microbial pathogens to avoid or reduce infectious disease outbreaks [139]. Next, nanocarriers enhance the natural compounds’ bioavailability and bioactivity by minimizing the nanoparticles’ size, surface modification, and encapsulating the natural compounds with different polymers [140]. Apart from the targeted drug delivery and improving the potential of the encapsulating drug, nanocarriers also protect the encapsulating natural bioactives from degradation, oxidation, and aggregation, which is considered an essential advantage in overseeing the use of nanocarriers over nanoparticles with surface fabrication [141]. Similarly, encapsulation using nanoparticles to transport natural drugs can improve the penetration of drugs into a biofilm and enhance either the removal of the biofilm or killing of the biofilm-encased pathogens. In addition, the toxicity of the drugs can be reduced by shielding its direct contact with the environment, and the drug’s enhanced stability can enable constant release over time [142].

##### Polymeric Nanoparticles

Polymeric nanoparticles are distinguished by their qualities customized for a specific payload and to the appropriate size, specific cellular trafficking, and easy regulation of drug delivery via improved material engineering. These polymeric materials are either natural (polysaccharides such as chitosan, cellulose, dextran, and others, or polypeptides and proteins, such as albumin, gelatin, legumin, and others) or synthetic (polyglycolide, polycaprolactone, derivatives of polyacrylic acid, poly(ethylene glycol), polylactides (PLA) and copolymers including polylactide co-glycolide (PLGA), and others) [143].

There are several reports on the potential of biopolymeric nanosystems against foodborne pathogens’ growth and biofilm formation (Table 4). These nanoparticles act via electrostatic interactions with the negatively charged EPS in the outer layer of the biofilms. Starch, cellulose, chitosan, cyclodextrin, alginate, and guar gum are a few examples of biopolymers often used to encapsulate natural products. Among these biopolymers, chitosan is a naturally occurring amino polysaccharide polymer with intrinsic antibacterial and antibiofilm properties. It has been a common biopolymer exploited to deliver natural products (Table 4) [144]. Earlier reports demonstrated that chitosan is a good choice of biopolymer for encapsulating natural compounds [145,146] and essential oils [147,148,149] against the biofilm formation of different foodborne pathogens. The use of chitosan nanoparticles is increasing in the active packaging sectors. In this light, a recent work by Khan et al. [150] reported that chitosan nanoparticles loaded with usnic acid, which is said to have antibiofilm activity against *S. aureus*, is efficient in eradicating the biofilm-residing persister cells of foodborne pathogens like *S. aureus*, *E. coli,* and *L. monocytogenes* [151]. Next to chitosan, another polysaccharide-based biopolymer that has been much exploited in drug delivery is cyclodextrin. Although three forms of cyclodextrins (α-, β-, and γ-cyclodextrins) are available, β-cyclodextrin is the most frequently used for encapsulation. Cyclodextrins are useful for encapsulating hydrophobic molecules, especially essential oils, as it contains both hydrophilic (outer) and hydrophobic (inner) parts [152]. The cyclodextrin and drug will form an inclusion complex through weak forces like van der Waals, hydrophobic, and hydrogen bonds. The antibiofilm drugs are encapsulated onto the cyclodextrin to make an inclusion complex for site-directed delivery. Several antibiofilm drugs have been encapsulated using cyclodextrin to eradicate the preformed biofilms of foodborne pathogenic bacteria [153,154,155].

Dendrimers are three-dimensional branched structures with repeated chemical patterns. It has attracted researchers in the field as it can encapsulate hydrophobic and hydrophilic compounds. Moreover, the addition of functional groups to the dendrimers increases the targeted delivery of drugs [156]. Studies have reported the delivery of some selected natural compounds such as resveratrol [157] and curcumin [158], against some human illnesses. Although dendrimers have long been applied in biomedicine, they have been recently discovered to act as antibacterial agents and coat surfaces [159]. However, there are reports of dendrimers encapsulating natural compounds against different infectious diseases. As stated above, the focus on the utility of dendrimers, especially against foodborne pathogens, and their biofilm-forming ability is on the rise. In this light, a recent study showed that the low molecular weight dendrimer peptides showed profound activity against foodborne pathogens such as *E. coli* and *S. aureus*, without incorporating any active drugs [160].

PLA and PLGA are the synthetic polymers that are most often used as nanocarriers. The use of these synthetic polymers has long been seen in different applications. Moreover, the Food and Drug Administration and European Medical Agency have approved these polymers for human use [161]. In this light, several works utilized these polymers to effectively deliver different antimicrobials against the growth and biofilm formation of foodborne bacteria (Table 4) [162]. However, their potential to carry and deliver antimicrobials inside the biofilms and eradicate the preformed biofilms is on the rise. In this light, a recent work by Anjum et al. studied the antibiofilm potential of xylitol when embedded in PLGA nanoparticles [163]. Xylitol cannot penetrate bacterial biofilms and is simultaneously degraded by the bacterial beta-lactamase enzymes. Incorporating xylitol into PLGA nanoparticles enhanced the penetration of xylitol into the EPS and resulted in subsequent biofilm disruption in *S. aureus*.

The surface charge of the nanoparticles plays a crucial role in their interaction with bacteria and biofilms. This was evidenced by the work of Da Costa et al. [164]. The authors modified the surface charge of the PLA nanoparticles by coating them with a positively charged peptide poly-L-lysine. These charge-reversed PLA nanoparticles had a pronounced biofilm-eradicating potential compared to nanoparticles with their original charge. The evidence for nanoparticles’ efficacy in halting bacterial pathogenesis is mounting. Further, to enhance the delivery of the antimicrobials or antibiofilm compounds into biofilms, a deeper understanding of the mechanisms governing the system’s efficacy is required, as shown by this study.

##### Lipid Nanoparticles

Lipid nanoparticles can be categorized as liposomes, nanoemulsions, solid lipid nanoparticles, and nanostructured lipid carriers. Due to their elastic physicochemical properties, well-established safety profiles, and ease of scaling up processes, lipid nanoparticles are some of the most promising tools for the targeted delivery of drugs [165]. Most lipids used as nanocarriers are approved by US Food and Drug Administration. Hence, the use of lipid nanoparticles is more prevalent than the previously described metallic and polymeric nanoparticles. Specifically, polyethylene glycol-grafted liposomes were in intelligent mode, which extended their presence for a longer time in the blood circulation without activating phagocytosis-mediated clearance or showing any toxicity [166]. Because of the above qualities, lipid-based nanoparticles have been extensively studied for targeting bacterial infections, especially against biofilm formation of bacterial pathogens (Table 4) [167]. In this regard, a recent study with nanostructured nano lipid carriers encapsulating olibanum oil was shown to kill sessile *C. jejuni* cells significantly more than the free oil [168].

Because of their industrial scale-up, biocompatibility, low toxicity, and ability to entrap both lipophilic and hydrophilic actives, liposomes are the most studied lipid-based nanoformulations (Table 4) [169]. Liposomes are spherical phospholipid bilayer vesicles. Liposomes preferentially adsorb onto biofilm surfaces and penetrate the EPS to prevent bacterial growth [170]. In addition to the advantages of using nanocarriers mentioned above, liposomes offer much more peculiar and advanced features, such as protecting volatile and chemically unstable antimicrobial or antibiofilm drugs (e.g., essential oils) against active component loss in air, light, and high temperature conditions during production, storage, and administration [171]. Specifically, soy lecithin and cholesterol (5:1)-derived liposomes extended cinnamon oil’s stability to 96 h and increased its antibiofilm activity against MRSA by almost ten times [171]. In light of developing drug resistance, liposomes entering the biofilm hide the drug from biofilm inhabitants until they burst. Thereby, the liposome prevents cells from recognizing the drug and developing resistance via horizontal gene transfer [172,173]. Apart from conventional liposomes, surface-modified liposomes such as PEG-derived liposomes, immunoliposomes, lectin-coated liposomes, and mannosylated liposomes, are reported to enhance the bioactivity of the encapsulating drug in eradicating and killing the biofilm and biofilm residents [173].

In addition to liposomes, antibiofilm lipid-based nanocarriers include nanoemulsions. Oil–water emulsifier nanoemulsions are isotropic and thermodynamically stable nanosystems. Their role as functional additives in different products such as cosmetics, topical drug delivery systems, and pharmaceuticals are promising. Nanoemulsions may penetrate porous matrices and touch the biofilm surface, enabling high antibacterial agent concentrations. Because of their adept penetration into porous matrices and intimate contact with the biofilm surface, nanoemulsions are very useful for disintegrating biofilms [174]. Lipophilic nanoemulsions interact with the EPS, thereby disrupting and disengaging the lipid layer [141]. A cumin oil-containing nanoemulsion was reported to target the biofilm formation in *E. coli* and *S. enterica* by reducing EPS production [175]. Moreover, the cumin oil-containing nanoemulsion significantly reduced the production of QS-related phenotypes in those foodborne pathogens. Similarly, another work by Prateeksha et al. [176] studied the potential of nanoemulsions of eugenol (0.0005%) and methyl salicylate (0.0025%) isolated from *Gaultheria fragrantissima* essential oil against the biofilm formation of *E. coli* O15:H7. The hydrogel containing the compound-loaded nanoemulsion reduced the surface colonization of *E. coli* on different surfaces. Furthermore, the essential oil nanoemulsion was reported to inhibit the biofilm-related genes in *E. coli*.

##### Silica Nanoparticles

Similar to other nanoparticles, silica nanoparticles can also encapsulate drugs. Often mesoporous nanoparticles are seen with the encapsulation of active leads after incorporating compounds with stimuli-based release properties [177]. Mesoporous silica nanoparticles are adaptable because pore size, particle size, and surface area can be easily modified. It also provides the prolonged release of encapsulated pharmaceuticals over hours or days [178]. In a study, mesoporous silica nanoparticles encapsulating different essential oils, such as eucalyptus, orange, and cinnamon, were employed to alter the adhesion, biofilm development, and preformed biofilms of *S. aureus* and *E. coli* [179]. The activity of the essential oil-encapsulated nanoparticles was found to be dependent on the phytochemicals used for encapsulation.

**Table 4:** Recent studies on the nanocarriers encapsulating natural products against the biofilms of foodborne pathogens.

Polymers Used for Nanoformulation	Natural Substance	Properties of Nanoparticles	Biological Activity against Foodborne Pathogens	References
Nanoparticles—biguanide-based polymetformin from Poly(ethylenimine)	Pluronic F-127 surfactant and tannic acid	Spherical-shaped nanoparticles with an average size of 96 nm and average zeta potential of -43 mV.	The biofilm-eradicating activity of nanoparticles plateaued from 8 µg/mL, which showed a 2.4 log_10_-fold biofilm reduction in MRSA. Along the same lines, the activity of biguanide-based polymetformin alone plateaued from 32 µg/mL with a 1.5 log_10_ reduction, and 256 µg/mL for vancomycin with a 1.1 log_10_ reduction. The nanoparticles showed good compatibility with human red blood cells, mouse embryonic fibroblast 3T3 cells, and human dermal fibroblast cells.	[180]
Nanoemulsion—Poly(oxanorbornenimide) modified with guanidinium, maleimide, and tetraethylene glycol monomethyl ether moieties	Eugenol, methyl eugenol, carvacrol, linalool, (+)-limonene, p-cymene, and α-pinene	The average size of the nanoemulsion containing different natural products ranged from ~180 to ~530 nm.	After 3 h of treatment, nanoemulsions with eugenol and carvacrol eradicated 90% of *S. aureus* biofilm. Compared to a carvacrol-loaded nanoemulsion, eugenol showed low toxicity to 3T3 fibroblast cells.	[181]
Polymeric Eudragit^®^ nanocapsules	Carvacrol	Nanocapsules with an average size of 156 nm, PDI value of 0.22, and zeta potential +44.8.	The nanocapsules were active in eradicating the biofilm formation of foodborne pathogens such as *Salmonella* spp. and *E. coli*.	[182]
Nanoliposomes (L80-T) and nanoarchaeosomes (A80-T)	Thymus vulgaris essential oil	Spherical vesicles. L80-T with Z potential −4.1 ± 0.6 mV, size ∼115 nm, and A80-T with Z potential −6.6 ± 1.5 mV and size ∼130 nm.	Compared to L80-T, A80-T was active against the preformed biofilms of *S. aureus* and its clinical isolates. Similar to the positive control vancomycin which has no effect even at a concentration 4 to 8-fold higher than the MIC_90_. However, A80-T at a concentration of MIC_90_ or MBC dislodged the preformed biofilms.	[183]
Liposomes	Berberine and curcumin	Spherical-shaped liposomes with an average size of 253 and a surface charge of −57 mV.	Compared to the free drug, co-encapsulation of BBR and CCR in liposomes decreased their MICs by 87% and 96%, respectively. At 10 µg/mL, dual drug-loaded liposomes disrupted the preformed biofilms of *S. aureus*. In addition, the dual drug-loaded liposomes reduced the bacterial burden and thereby the infection in L929 fibroblast cells.	[184]
Liposomes	Antilisterial peptide (Lys-Val-Asp-His-Phe-Pro-Leu) originated from rice bran protein	Spherical-shaped liposome with an average size of 137.9 ± 3.1 nm and a surface charge of 28.9 ± 0.8mV.	The MIC of liposomes with antilisterial peptide (84.26 µg/mL) against the sessile *L. monocytogenes* cells was higher than the free peptide (8 µg/mL). However, the liposome encapsulating peptide showed a rapid release and killing of *L. monocytogenes* cells after 10 min of contact time, which was 120 minutes for free peptide. The liposome-encapsulated drug showed flavor stability for four weeks when stored at 4 °C.	[185]
Sol-gel–derived from tetramethylorthosilicate	Garlic extract	--	Garlic extract-loaded nanoparticles were efficient in penetrating and disrupting the well-established MRSA biofilms. At 5mg/mL, the extract-loaded nanoparticles reduced the biofilm-residing bacterial viability to 80% and reduced the bacterial biofilm thickness to 2.3 µm from 9.6 µm (control).	[186]
Solid lipid nanoparticles–Chinese white wax	Curcumin	Spherical-shaped nanoparticles with an average particle size of 402 nm.	The curcumin-loaded nanoparticles showed a sustained drug release profile and showed a higher drug release rate under an acidic (pH 4.5) rather than a neutral (pH 7.4) environment. The curcumin-loaded nanoparticles inhibited the biofilm formation of *S. aureus* at a concentration of 125 µg/mL.	[187]
Solid lipid nanoparticles	Cinnamon oil	Spherical-shaped nanoparticles with an average size of 337.6 nm and zeta potential of −26.6 mV.	Encapsulated solid lipid nanoparticles reduced the antimicrobial activity against *E. coli* by 2-fold compared to the cinnamon oil alone. Further, the sub-MIC of solid lipid nanoparticles reduced biofilm formation by 55.25%.	[188]
Starch nanoparticles	Triphala formulation containing the blended extract of *Terminalia chebula*, *Terminalia bellirica*, and *Emblica officinalis*	The average size of the nanoparticle was 283 nm with a zeta potential of −12 mV.	The starch nanoparticle-encapsulated drug showed a dose-dependent antibiofilm activity against MRSA, with 10 µg/mL being the most effective biofilm inhibitory concentration.	[189]
Solid lipid nanoparticles - Glyceryl monostearate and Poloxamer 188	Anacardic acid	The average size of the nanoparticle was 212 nm with zeta potential and PDI value of−13 mV and 0.285, respectively.	Treatment with anacardic acid-loaded nanoparticles reduced the biofilm formation of *S. aureus* at 0.097 µg/mL. The nanoparticle treatment reduced the biofilm thickness (50.3 µm, in control) and biofilm biomass (27.77 μm^3^/μm^2^, in control) to 18.61 µm and 10.54 μm^3^/μm^2^, respectively.	[190]
Chitosan nanoparticles	Chrysin	Spherical-shaped nanoparticles with an average size of 355 nm and with PDI value of 0.487.	Chrysin-loaded nanoparticles inhibited the biofilm formation of *S. aureus* to 54% at 768 µg/mL. The nanoparticles also reduced biofilm-related phenotypes, such as EPS and cell surface hydrophobicity.	[145]
Chitosan nanoemulsion	Thymol/thymol essential oil	The mean particle size of thymol and thymol essential oil were 123 and 139 nm, respectively.	Compared to the thymol essential oil, thymol in a nanoemulsion showed better biofilm inhibition against *S. aureus* (83.78%) and *E. coli* (83.64%). The thymol nanoemulsion enhanced the shelf life by reducing the total viable count in the pork sample.	[147]
Chitosan nanoparticles	*Citrus reticulata* essential oil	The average particle size ranges between 131 and 162 nm.	The activity of encapsulated nanoparticles was based on the concentration of *C. reticulata* essential oil. Chitosan nanoparticles loaded with equal volumes of *C. reticulata* essential oil showed good biofilm inhibition and eradication activity against *S. aureus* and *E. coli*.	[148]
Chitosan nanoparticles	Cinnamaldehyde	The average size was 298.1 nm with a zeta potential of +38.73 mV.	Cinnamaldehyde-loaded chitosan nanoparticles at 1.25 mg/mL showed antibiofilm activity. Further, these nanoparticles eradicate the preformed biofilm of *S. aureus* to 48.1%.	[146]
Chitosan	*Prangos acaulis*	Semi-spherical nanoparticles with an average size of 89.8 nm and a zeta potential of 10.78 mV.	The chitosan nanoparticles were effective against the Gram-positive bacteria *S. aureus* and *B. cereus* with a biofilm inhibitory rate of nearly 50% compared to the Gram-negative bacteria *E. coli* with a biofilm inhibitory rate of 32% at 2000 µg/mL.	[191]
PLGA	Xylitol	Nanoparticles with an average size of 106–140 nm and a zeta potential of 12.29–34.05 mV.	PLGA nanoparticles loaded with 5% xylitol showed a profound preformed biofilm-eradicating and killing activity against *S. aureus*. The biofilm penetrating potential of PLGA nanoparticles enhanced the biofilm-eradicating ability of xylitol.	[163]
Nanoemulsions	Cinnamon and clove essential oils	The average size of the nanoemulsion was less than 15 nm. The PDI and zeta potential values were below 0.37 and −10.5 mV, respectively.	Compared to Brij-35, the nanoemulsion formed using Tween-20 showed an enhanced rate of biofilm inhibition of 76% in *S. aureus*.	[192]
Nanoemulsions	Eugenol and methyl salicylate	The mean diameter of eugenol and methyl salicylate was 9.389 ± 0.2 nm and 10.81 ± 0.4 nm, with PDI values of 0.345 and 0.371, respectively.	The nanoemulsion carrying the drugs showed higher antibiofilm and antivirulence activity against *E. coli* O157:H7. The expression of genes related to the biofilm formation in *E. coli* was significantly downregulated upon treatment with the drug-loaded nanoemulsion.	[174]
Nanostructured lipid carriers	Olibanum oil	The resultant nanoparticles had a particle size of ~200 nm, a polydispersity index of ~0.15, and a zeta potential of ~−35mV.	The nanostructured lipid carriers encapsulating olibanum oil effectively killed the biofilm resident *C. jejuni* cells and had an MIC value of 780 µg/mL, which was very significant compared to the free oil with an MIC of 1–3 mg/mL.	[168]

## 5. Strategies to Enhance Biofilm Clearance

Compared to free compounds, NPs exhibit increased efficacy in preventing biofilm formation and eliminating mature biofilms (Table 4). In general, antibiofilm activity can be achieved by modifying the surface properties of the nanoparticles, which target bacterial adherence and eliminate the adhering bacterial pathogens. Although the complete eradication of mature biofilms is challenging, it has been accomplished in rare instances. It is believed that at most moments, the cargo was deactivated either by the harbored enzyme or the microenvironment. With antibiotics loaded in lipid liquid crystal nanoparticles, 100 percent biofilm eradication was observed, although a 3-fold greater concentration of free antibiotic failed to eliminate the preformed biofilms [193].

In contrast, a few nanoparticles were ineffective. For instance, nanoparticles showed MIC values at the microgram level or failed to eradicate preformed biofilms or kill the biofilm residents [145,191,194]. Zein nanoparticles loaded with anacardic acid could not kill and destroy the residents of preformed biofilms [195]. Similarly, liposomal-loaded antilisterial peptide showed a 10-fold increased MIC and antibiofilm inhibition compared to the free peptide [185]. This was attributed either to the low drug delivery inside the biofilm or to the inability of the nanoparticles to penetrate the biofilm matrix. Hence, it is crucial to determine appropriate methods to boost NPs’ antibiofilm abilities.

### 5.1. Size and Surface Modifications

The capacity of nanoparticles to penetrate biofilms is directly related to their size. Depending upon the density of the EPS matrix, water channels and meshes in biofilms vary in size from 10 nm to hundreds of nm [196]. The diffusion rate of nanoparticles within a biofilm is also directly proportional to its size [197,198]. It is suggested that nanoparticles with an average size of 130 nm or smaller can penetrate the biofilm matrix and have better biofilm-eradicating potential [199]. However, most nanoparticles with this cutoff are attributed to the metallic nanoparticles and necessitate the reassessment of those nanoparticles with small sizes for biofilm penetration and eradication. Notably, the nanoparticles with this cutoff are primarily associated with the metallic ones (Table 3). In contrast, organic NPs are malleable and biodegradable and must be validated whether the observed cutoff value applies to them.

Similar to size, surface properties of the nanoparticles, like surface charge and hydrophobicity, are also essential factors determining their biofilm penetrating potential. Coating PLA nanoparticles with the positively charged peptide poly-L-lysine enhanced biofilm-eradicating capacity compared to uncharged nanoparticles [164]. The cationic polymer chitosan is predicted to prevent bacterial biofilm formation by reacting electrostatically with the negatively charged EPS, proteins, and DNA that make up biofilms [144,200]. Moreover, nanoparticles engineered with biofilm-degrading components such as DNase, proteinases, and β-N-acetyl-glucosaminidase, showed better biofilm-eradicating potential [201,202]. Other positively charged nanoparticles show better binding and penetrating potential, possibly through electrostatic interaction with biofilm components [146,191,203]. However, a deeper study is required to determine whether this is the actual scenario. Similarly, hydrophilic nanoparticles with a negative or neutral charge are suitable for the penetration of nanoparticles intended for mucosal delivery [204] but can also be applied for biofilm penetration.

### 5.2. Stimuli-Responsive Release 

In the presence of external cues, it is possible to create nanoparticles that trigger the release of encapsulated drugs. An attraction-luring, intelligent, and potential therapeutic technique for optimized drug release is using stimuli-responsive drug delivery systems that are sensitive to a wide range of endogenous stimuli including pH, redox state, and temperature. In this light, the acidic pH of the biofilm can be used as the external stimulus to trigger the release of the encapsulated drug. Cationic farnesol-loaded nanoparticles were synthesized using the co-polymers 2-(dimethylamino) ethyl methacrylate, butyl methacrylate, and 2-propylacrylic acid [205]. As a cationic nanoparticle, it showed a high affinity towards biofilm components. Moreover, in the acidic biofilm environment, the pH-sensitive core accelerated the sustained release of farnesol into the biofilm for a more extended period. Compared to the free drug (20%), the farnesol-loaded nanoparticles reduced the preformed bacterial biofilm by 80% [206]. In addition, the farnesol-loaded nanoparticles attenuated the infection in vivo. The nanoparticles loaded with saturated farnesol enhanced biofilm eradication by 3-fold and 7-fold compared to the farnesol-loaded nanoparticles and farnesol alone [207].

Similar to pH, the enzymes secreted by bacteria can also be an external cue that can be used to trigger drug release. Specifically, hyaluronidase [207], lipase [208], gelatinase [209], and glutamyl endonuclease [209] have also been used as enzyme cues for triggered drug release. Wang and Shukla synthesized an antibiotic nanoparticle loaded on a gelatin core and coated with chitosan and hyaluronic acid [207]. Hyaluronidase produced by the bacteria first degraded the hyaluronic acid, exposing the chitosan coating, which in turn aids enhanced biofilm adhesion and penetration. As the chitosan layer expands, more gelatinases could reach the inner core, leading to gelatin breakdown and drug release [206]. Compared to the free drug, the drug loaded in the nanoparticle coated with hyaluronic acid enhanced the eradication of the biofilm.

In another work, Wu et al. [177] synthesized mesoporous silica nanoparticles decorated layer by layer with stimuli-responsive materials. The lysozyme and amoxicillin encapsulated with mesoporous nanoparticles were first coated with 1,2-ethanediamine, a cationic polymer that shows non-specific electrostatic interactions with bacterial membranes, and then with hyaluronic acid on which the hyaluronidase can act. They demonstrated the systemic release of lysozyme and amoxicillin and the antibacterial potential of the synthesized nanoparticles against *E. coli* and *S. aureus* [177], highlighting the use of a combination of stimuli-responsive materials in the drug delivery system for the eradication and killing of biofilm cells.

Apart from the endogenous cues, targeted drug delivery is possible using endogenous signals, such as magnetic fields, ultrasound, electric fields, and light [210,211,212]. Recent strategies included the use of ultrasound. Along with the nanoparticle, the use of ultrasound effectively enhanced the eradication of biofilm formation. Recently, Gopalakrishnan et al. [213] reported using a combination treatment involving antimicrobial polymeric nanoparticles and ultrasound. The ultrasound treatment dislodged the preformed biofilms and enhanced the penetration of the polymeric nanoparticles. This combined treatment strategy improved biofilm eradication by 100- to 1000-fold without showing toxicity to fibroblast cells.

As a very affordable and accessible external stimulus, light is widely used for highly precise, controlled drug release from responsive nanocarriers [214]. In particular, light in the near-infrared region is appropriate for treating biofilm infections because of its longer wavelength, greater tissue penetration, and low toxicity. In addition, near-infrared region light may operate as a thermal trigger since photothermal agents can convert it to heat. This conversion is often utilized in materials that respond to light since light is considerably simpler to regulate than heat [214]. In a study by Zhao et al. [215], liposomes encapsulated with the near-infrared light-responsive agent cypate and antibiotic eliminated biofilm formation by pathogenic bacteria. The liposomes remained stable at room temperature. Above 40 °C, the liposomes released more than 80% of the antibiotics into the medium. The synthesized nanoparticles showed a more than 80% biofilm reduction, which was 8-fold higher than the free antibiotic alone.

Like near-infrared light, magnetic fields are also widely used in medicine. Magnetic nanoparticles can target particular biofilm infection sites and penetrate protective biofilm matrices with the energy from external magnetic fields. In addition, they have been shown to induce local heat and mechanical stresses, which have the potential to deteriorate polymeric materials and result in drug release. In their study, Yu et al. assessed the activity of two co-assembled mesoporous silica nanoparticles containing antimicrobial compounds [216]. The melittin-containing mesoporous nanoparticle was capped with β-cyclodextrin modified with polyethylenimine (Host-MSN). The other magnetic nanoparticles contained ofloxacin and were decorated with adamantane and capped with curcubit[6]uril (Guest-MSN). Compared to the free drugs, the conjugated nanoparticles eradicated the biofilm of the pathogenic bacteria by removing the biofilm biomass and rapidly killing biofilm residents. Furthermore, these nanoparticle co-assemblies exhibited no toxicity to mammalian cells and prevented biofilm formation in vivo without causing host tissue damage and inflammation.

### 5.3. Combined Strategies 

The combination of two different antibiofilm strategies will have synergistic activity by enhancing biofilm eradication. The innovative nanoencapsulation approach of liposome co-encapsulating antibiotics with metals improves their antibacterial and antibiofilm properties. For instance, gallium targets iron metabolism and iron-dependent cellular processes to impede bacterial growth and biofilm formation [217,218]. Following this approach, the growth and biofilm formation of *P. aeruginosa* was targeted by DPPC/dipalmitoyl phosphatidylglycerol (DPPG) liposomes containing gallium and gentamicin. This formulation outperformed liposome-loaded gentamicin and the free antibiotic against planktonic and biofilm *P. aeruginosa* cells. This metal–antibiotic-loaded liposome also eliminated bacterial biofilms and QS signaling and lowered gallium toxicity [219]. Bismuth and bismuth-thiol, like gallium, alter iron uptake, alginate expression, lipopolysaccharides, virulence factors, bacterial adhesion, and biofilm formation to inhibit a broad spectrum of microorganisms [220,221,222,223]. Hereto, the liposome co-encapsulated with tobramycin and bismuth-ethanedithiol was effective against *Burkholderia cepacia* and *P. aeruginosa*. The drug-free liposomes with bismuth-ethanedithiol and the free drug had significantly reduced MIC and MBC values. Metal and drug co-loaded liposomes decreased A549 human lung cancer cell toxicity compared to free bismuth-ethanedithiol and tobramycin. In addition, the liposomes co-encapsulated with tobramycin and bismuth-ethanedithiol reduced biofilm-forming *P. aeruginosa* growth at 0.064 mg/L and suppressed QS below the free drug and bismuth MICs. Bismuth-ethanedithiol tobramycin, bismuth-ethanedithiol-tobramycin, liposomal tobramycin, and co-encapsulated with tobramycin and bismuth-ethanedithiol did not perform better individually [224].

Similar to co-encapsulation, it is possible to synthesize a mixed nanohybrid system involving both polymers and lipids. This nanohybrid system has the advantages of biocompatibility and enhanced drug delivery, which the lipids and polymers will provide, respectively. This was substantiated by the work of Gou and colleagues [225], wherein this nanohybrid system boosted antibiotic penetration inside the biofilm and improved the treatment of MRSA infection.

Similarly, the simultaneous construction of the size and pH-responsive nanoparticles enhanced biofilm eradication [226]. Azithromycin-conjugated amino-ended poly(amidoamine) dendrimer- and 2,3-dimethyl maleic anhydride-modified PEFG-block-polylysine were synthesized and assessed for their biofilm-eradicating potential [35]. The acidic biofilm environment disassembles the primary structure of the nanoparticles, leading to the release of the antibiotic-conjugated dendrimer, which is small and has a better biofilm penetrating potential. The pH-responsive nanoparticle efficiently reduced bacterial burden and inflammation in a lung infection model. A combination of enzyme modification and photothermal therapy alleviated MRSA biofilms [227]. A mesoporous polydopamine nanoparticle was fabricated with near-infrared-responsive carbon monoxide and biofilm-disrupting enzyme DNase. The release of DNase destroyed the rigidity of the biofilm. After near-infrared light irradiation, the nanoparticles released antibacterial carbon monoxide on the impaired biofilms [227].

Antimicrobial photodynamic therapy (aPDT) eliminates specific cells by inducing reactive oxygen species. This approach utilizes three different factors such as light (at an appropriate wavelength), a photosensitizer (a compound activated by light), and molecular oxygen (for the generation of ROS) [228,229]. Singlet oxygen, produced explicitly by this approach, triggers cell death by interacting with practically all cellular components and biomolecules. Among several synthetic photosensitizers, natural compounds such as curcumin, hypericin, and flavin derivatives, are used [229]. A study by Cossu et al. [230] demonstrated the synergistic activity of gallic acid and UV-A against the biofilm formation of *E. coli* O15:H7. This synergistic approach reduced the metabolic activity of the cells residing in biofilms by 80% when exposed to 30 min of UV-A with 10 mM gallic acid. Similarly, the photosensitizers encapsulated with mesoporous silica nanoparticles reduced EPS production and biofilm formation by *S. aureus* and *P. aeruginosa* [231]. Considering the role of aPDT against the resistance development among foodborne pathogens, the report of Al-Mutairi et al. [232] in 2018 stated that aPDT is not supposed to induce antimicrobial resistance in bacterial pathogens.

## 6. Conclusions and Future Perspectives

As detailed above, a surplus of clean labeled compounds has been identified from natural resources with growth- and biofilm-inhibiting and biofilm eradicating potentials against foodborne bacterial pathogens. However, certain limitations hold back the applications of these identified natural products. For instance, the identified bioactive might have low bioavailability, bioactivity, solubility, and stability. Moreover, microbial biofilms with altered microenvironments might modify the functions of natural bioactive compounds. Encapsulation or fabrication of natural bioactives with nanoparticles will enhance the activity compared to the free drugs in most instances.

To conclude, this review has summarized the most recent works that utilize the nanotechnology approach to enhance the potential of natural products in the combat against the biofilms of foodborne bacterial pathogens. The use of antibiofilm nanoparticles in food sectors is in its infancy. However, the rise in studies over the last few years indicates the topic’s increasing significance.

Although nanoparticles loaded with natural products show superior abilities, several issues need to be resolved when these nanoparticles are used in foods: (1) future studies focusing on the impact of nanoparticles on biofilms. Using natural products in nanoparticles shows some undesired effects [185]. However, insufficient knowledge of the properties of nanoparticles and the drug being encapsulated is thought to be the primary cause of those effects. Recent studies insist on the importance of nanoparticle properties such as size, shape, and surface charges, in biofilm studies [164,199,233]. However, the interaction studies dealing with nanoparticles and biofilm matrix components are minimal and remain a pivotal question to be explored [197,234]. (2) More studies focusing on the impact of food and food components on nanoparticles need to be conducted. The activity of nanoparticles depends on the nature and the load of the drug that is being encapsulated. Natural products are extremely susceptible to degrading enzymes and environments with altered pH and temperature. However, these can be overcome with specific strategies like a stimuli-based release. (3) Studies need to be performed focusing on the toxicity of the nanoparticles and the detailed evaluation of critical aspects such as toxicokinetics, translocation, and synchronized response of different tissues to the nanoparticles. In this regard, the drug-loaded nanoparticles have mostly been studied in vitro, and little is known about their potential in animal models. However, some nanoformulations have shown promising results in animal models, namely, silver [119], copper [127,235,236], magnesium oxide [237], polymeric [180], and silica nanoparticles [216]. Meanwhile, the use of drug-loaded nanoparticles in food systems has gained considerable attention from regulatory boards regarding the consumers’ health after ingesting food products with residual nanoparticles [238]. In addition, they can surpass biological barriers and lead to bioaccumulation in various organs, tissues, and cells [239]. The World Health Organization and U.S. Food and Drug Administration’s Nanotechnology Task Force was established to evaluate the risks, explore nanomaterial qualities, and draft stringent rules to govern their usage [238]. However, no standard for health risk assessments has been developed for these nanomaterials before their approval for use in foods. Nevertheless, a comprehensive evaluation of these aspects needs further research. (4) Finally, the studies focusing on application-oriented research is needed. To overcome the limitations in the applicability of antibiofilm nanoparticles, the concurrent advances in active packaging technologies can also be expected to contribute to realize the promise of safe and effective usage of nanoparticles in food systems [240]. Keeping the advantages and disadvantages in mind, future efforts in the standardization and the assessment of nanoformulations in food will enhance the use of natural products in the battle against biofilms of foodborne bacterial pathogens.

## Figures and Tables

**Table 1:** Selected foodborne pathogens with biofilm-forming ability in the food industry.

Foodborne Bacterial Pathogens	Selected Important Pathogenic Attributes and Essential Genes or Proteins Involved in Biofilm Formation	Favorable Conditions and Frequently Associated Food Products	Diseases Conditions	References
*L. monocytogenes*	Listerial adhesive proteins mediate adherence in *L. monocytogenes*. Further, internalins and phospholipases ensure their internalization in host cells. In addition, it produces other proteins like listeriolysin (causing infection) and actins (aiding mobility between host cells). Putative response regulator (DegU), flagellar proteins (FlaA and Mot), quorum sensing proteins (AgrBDCA), biofilm-associated protein (BapL), adhesin proteins (LAP), D-alanylation pathway (DtlABCD), internalins, phospholipases, phosphate-sensing two-component system (PhoPR), autoinducer-2 (LuxS), autolysin, SigB, *relA*, *hpt*, and SecA2 are reported to be involved in biofilm formation.	It can survive in harsh environments, such as varied pH (4.0–9.6) and temperature (−1.5 to 45 °C). It is frequently associated with dairy products, fresh produce, and ready-to-eat foods.	Ingestion of contaminated food leads to gastroenteritis or Invasive listeriosis. The invasive systemic disease targets immunocompromised hosts and lead to miscarriage and stillbirth in pregnant women. It can cause meningitis or encephalitis in newborns and the elderly.	[15,16,17]
*Salmonella* spp.	Adherence is mediated by the expression of proteins related to motility (fimbriae and flagella) and different adhesion proteins (BapA, SiiE, ShdA, MisL, and SadA). Bap and curli (CsgD) and cellulose (BcsA) biosynthesis are reported to be involved in biofilm formation.	It can grow at temperatures ranging between 8 and 45 °C and at a pH ranging between 4.0 and 9.5. Incidence, dissemination, and persistence of infections are often observed when consuming raw foods like fruits and vegetables.	*Salmonella* infection, often known as salmonellosis, is characterized by an early onset of fever, diarrhea, and stomach cramps within 12 to 72 h after exposure.	[18,19]
*E. coli*	Molecular determining factors responsible for surface colonization include exopolysaccharides, lipopolysaccharides, poly-N-acetyl glucosamine, colonic acid, lipoproteins (SslE), *E. coli* factor adherence 1 (Efa1), immunoglobulin binding protein (Eib), and fimbrial (AAF, Fim) adhesins. It also produces heat-stable (STa, STb) and heat-labile (LTp/h, LT-IIa, LT-IIb) enterotoxins.	*E. coli* thrives in aerobic and anaerobic conditions and temperatures ranging from 23 to 40 °C and at a pH of 6.5 to 7.5. Contaminated ground meat, dairy, and fresh vegetables are the causes of most outbreaks.	*E. coli*, a sign of feces in food and water, suggests inadequate hygiene. Food infected with heat-stable enterotoxins from pathogenic *E. coli* may induce food poisoning.	[20,21]
*S. aureus*	Produce enzymes that degrade phospholipids, elastin, DNA, and hyaluronic acid, and facilitate tissue penetration. Express different pore-forming toxins to evade host immune response, such as leukocidins, phenol-soluble modulins, protein A, Eap, staphyloxanthin, staphylococcal complement inhibitor, and capsular polysaccharides. Express toxins like Toxic shock syndrome toxin-1, α-toxin, enterotoxins, exfoliative toxins A and B, and lipoteichoic acid. Microbial surface components that recognize adhesive matrix molecules include surface proteins such as bone sialoprotein-binding protein, fibronectin-binding proteins, clumping factors, and fibrinogen-binding protein. Produce several proteins involved in biofilm formation, such as SasG, CidA, Bap, Spa, and polysaccharide intracellular adhesions (*ica* operon).	Grow at a temperature ranging from 15 to 45 °C, with pH ranging between 4.0 and 9.8. Staphylococcal food poisoning happens with consuming dairy products from animals suffering from *S. aureus* mastitis. In addition, there are a lot of people who carry the bacteria. This is primarily because of improper food handling.	Food pathogenic *S. aureus* strains produce enterotoxins. Due to their stability, heat tolerance, and capacity to withstand freezing and drying, these enterotoxins are a significant food business risk. These enterotoxins cause staphylococcal food poisoning outbreaks when consumed.	[22,23]
*Campylobacter* spp.	Express CadF, JlpA, PEB1, and CapA for different surface adherence. Express a wide range of flagellar protein required for host cell adhesion, biofilm formation, and secretion of invasive proteins that suppresses the host immune response. In addition, they produce toxins (CdtA, CdtB, and CdtC).	Grow between 37 °C and 42 °C (optimally at 41.5 °C), with fresh chicken meat as the most implicated food type.	Among the *Campylobacter* spp., *C. jejuni* and *C. coli* are the most significant. Campylobacteriosis causes diarrhea, stomach discomfort, fever, headache, nausea, and vomiting	[24,25]

## Data Availability

The data from the reviewed articles can be found in Pubmed (https://pubmed.ncbi.nlm.nih.gov/, accessed on 30 December 2022) and Google Scholar (https://scholar.google.com/ accessed on 30 December 2022).
